# Mortality among adolescents and young adults in specialized substance use treatment: a Swedish register study

**DOI:** 10.1186/s13034-026-01125-1

**Published:** 2026-06-24

**Authors:** Patrik Karlsson, Mats Ekendahl, Philip Lindner

**Affiliations:** 1 Department of Social Work, Stockholm University, Albanovägen 18, Stockholm, 106 91 Sweden; 2 Centre for Psychiatry Research, Department of Clinical Neuroscience, Karolinska Institutet, & Stockholm Health Care Services, Region Stockholm, Stockholm, Sweden; 3 Stockholm Centre for Dependency Disorders, Stockholm Health Care Services, Region Stockholm, Stockholm, Sweden

**Keywords:** Substance use treatment, Adolescents, Young adults, Mortality, Sweden

## Abstract

**Background:**

Individuals with substance use problems have higher mortality risks than the general population. This study aimed to assess all-cause and specific-cause mortality among adolescents and young adults in specialized substance use treatment in Sweden, a largely overlooked group in the literature.

**Methods:**

A registry cohort was compiled of patients (13–25 years) who between 2011 and 2021 were in contact with Sweden’s largest healthcare provider of adolescent substance use treatment, with mortality data covering the same period. Sex, migrant background, parental education, convictions and treatment variables were compiled from registries and included as predictors. We calculated crude mortality rates (CMRs) and estimated Cox regression models to explore the associations between the predictors and mortality. This also included testing for interactions between convictions and treatment variables.

**Results:**

Among 23,709 patients, 229 deaths were recorded, meaning that slightly less than 1% died during the follow-up window. The overall CMR was 1.68 deaths per 1000 person years (95% CI = 1.47–1.91), with differences across some predictors. In the fully adjusted Cox regression, significantly elevated mortality risks were observed among males (HR = 1.69, 95%CI = 1.23–2.32), those who had received inpatient treatment (HR = 1.89, 95% CI = 1.42–2.52), and those who had been convicted (HR = 2.64, 95%CI = 1.92–3.63). No significant difference in mortality risk was observed for migrant status, parental education, or number of outpatient visits in the fully adjusted models. Among convicted patients, more outpatient visits were related to a lower mortality risk (HR = 0.98, 95%CI = 0.98–0.99), whereas inpatient treatment was associated with a higher mortality risk (HR = 2.14, 95%CI = 1.13–4.05).

**Conclusions:**

Almost one out of hundred patients died during the follow-up period, greatly exceeding the mortality rate in adolescents and young adults in the general Swedish population. The mortality risk varied according to sex, treatment experiences, and convictions, but not according to migrant background or parental education. In sum, we report a complex relation between convictions, treatment, and excess mortality among adolescents and young adults enrolled in specialized substance use treatment, signaling profound unmet clinical and social work needs.

**Supplementary Information:**

The online version contains supplementary material available at 10.1186/s13034-026-01125-1.

## Introduction

Adolescent substance use (ASU) is associated with a number of negative outcomes, of which mortality is the ultimate. Substance use problems, together with mental health problems, is the leading contributor to disability among adolescents in high income-countries and account for a substantial number of deaths worldwide [1]. In the US, due to the opioid-epidemic, drug related deaths among young people are of particular and rising concern [2, 3], but the issue has also been discussed in the context of Sweden [4], where drug-related deaths are the second and the third most common preventable cause of death among 18–49-year-old males and females. While suicide has remained the most common cause of death [5], the past decade has witnessed rising numbers of drug-related deaths in the country [6].

The observed mortality risk is particularly elevated among adolescent and adult patients in mental health and substance use treatment [7–14], a link reasonably explained by clinical severity. In fact, compared to the general population in the same ages, standardized mortality rates (SMRs) are higher in younger than in older age groups with substance use problems, particularly among those who have been convicted for both non-violent and violent crimes [10]. There are also indications that mortality rates are more elevated among adolescents in substance use treatment compared to adolescents in mental health treatment [15], and substance use problems have been linked to higher SMRs than many other psychiatric conditions [16]. While it is difficult to disentangle the impact of treatment as such from the number of factors that push people into it, the mortality risk remains high for many years following treatment admission [17].

However, there is limited recent research on mortality and its predictors among adolescents and young adults in treatment [15, 18], with most research focusing on adult samples [19–21]. Adolescents typically present a different clinical profile [18] and are less likely to seek help on their own accord [22]. Younger substance use treatment populations are also more likely to die from drug poisonings and suicides compared to their older counterparts [23]. While there are some previous Swedish studies on mortality among patients who as adolescents attended specialized substance use treatment between the 1960s and the 1980s [24, 25], there have since been dramatic changes in both adolescent substance use patterns in general [26], as well as in the organization and practice of adolescent substance use treatment. Importantly, Sweden has during the past decade experienced increased rates of deadly violence related to organized crime, usually committed by and targeted at adolescents and young adults who are involved in illegal drug markets [27]. In media and politics, this violence has been attributed to young people with immigrant backgrounds in so called exposed areas [28], making it important to examine whether this development has rendered past associations obsolete. While the broader mortality literature has increasingly addressed drug overdoses, alcohol-related deaths, and suicides – collectively referred to as “deaths of despair” – for understanding trends in mortality rates (see e.g. [29–31]), – the upsurge in violent deaths is an increasingly important factor to consider in the Swedish context.

The aim of the current study was to explore mortality among adolescents and young adults (13–25 years of age) who were in contact with Sweden’s largest healthcare provider of specialized adolescent substance use treatment between 2011 and 2021. We primarily focus on all-cause mortality, but also on cause-specific mortality related to drug and alcohol-related deaths, suicides, and homicides. Predictors include socio-demographics (sex, migrant background, parental education), treatment variables (outpatient and inpatient), and convictions. While socio-demographics are standard in mortality studies, with some exceptions [32], larger register studies typically do not include treatment variables. Convictions, in turn, have been of central concern in mortality research on both general population and clinical samples [10, 33–35]. Studies on criminal justice samples have identified an excess mortality risk among users of certain substances [36], suggesting that substance use problems and criminality may interact in their association with mortality. Here, we exploratively test for interaction between treatment experiences and convictions. Convictions may be a proxy for risk behaviors [10] and as amount of treatment received may indicate problem burden related to substance use, testing whether treatment moderates the association between criminality and mortality is important for identifying patient groups at particular risk.

## Data and methods

### Sample

The initial sample comprised all patients who according to electronic health records were admitted to the Maria Ungdom (MU) Stockholm clinic between January 1 2011 and December 31 2021 (n = 31504). MU Stockholm is Sweden’ largest provider of regional (i.e., healthcare), specialized substance use treatment for adolescents and young adults aged 13–25 years. At current, MU Stockholm consists of 24 treatment clinics located in the Stockholm region. It includes one inpatient ward, three larger outpatient clinics, and around 20 smaller, local clinics. Patients may be enrolled in care for a number of reasons, including but not limited to referrals from child and adolescent psychiatry, social services, schools or parents, but also because of acute intoxication. Most patients receive outpatient treatment only, and all those who first receive inpatient treatment are also offered outpatient treatment subsequently. During outpatient contacts, intermixed inpatient visits, both planned and non-planned, are not uncommon, for example due to acute intoxication, or if abstinence is required for change in medication or for assessment purposes. Alongside immediate and short-term acute detoxification care equivalent to what the Stockholm Addiction Emergency Ward offers to adult patients [37], admittance-based inpatient care typically has 7 ± 2 days duration and may include medically managed detoxification if necessary, but typically focuses on offering a supportive and controlled environment for stabilization. As is distinctive of Swedish healthcare, participating in offered healthcare, both outpatient and inpatient, is voluntary (unless rare exceptions apply), although it is well-known that other actors (e.g., social services) may condition their help on participation in healthcare. Thus, some patients may perceive their treatment at MU as de facto mandatory. During the covered period, no substantial changes occurred in the organization of MU Stockholm [38].

In this study, patients who according to the records were not aged 13–25 at first contact (inpatient or outpatient, whichever occurred first) with MU Stockholm were removed from analysis (*n* = 1533) as they either were not typical patients (e.g., 11 years old) or because of apparent errors in age records (e.g., 70 years old). A few duplicates (*n* = 8) and two patients who did not have their first contact between 2011 and 2021 were removed. Six additional patients were excluded because of insufficient data regarding their death dates. In all, also due to missing data on country of birth (*n* = 1402), maternal education (*n* = 3886), and paternal education (*n* = 5602), the final sample comprised 23,709 patients. This comprises 79.1% of all patients 13–25 years who had their first contact with MU between 2011 and 2021.

### Outcome variables

The primary outcome variable was all-cause mortality during the period 2011 and 2021. Data on deaths were obtained by linking the patients’ personal numbers with the Swedish National Board of Health and Welfare’s Causes of Death Register. The register includes all deaths in Sweden, including information on death date, the ultimate cause of the death, and contributing causes. In addition to all-cause mortality, prevalence of select causes of death were also analyzed (see Supplementary file 1 for definitions).

### Predictors

We included several predictors related to treatment and socio-demographics. Treatment-related variables were derived from the patients’ electronic health records, compiled using a database query: number of outpatient visits and an indicator of whether patients had received inpatient treatment or not.

An indicator of whether the patients had been convicted during the period was also included. In Sweden, the age of criminal responsibility is 15, and data on convictions between 2011 and 2021 were compiled from the conviction register, held by the Swedish National Council for Crime Prevention. Patients with at least one conviction record were distinguished from patients with no conviction record, but we did not make any further comparisons in mortality according to the main offense included in the conviction or type of sentence (e.g., fine or prison). While it would have been possible to construct variables with higher levels of detail, we chose a binary indicator to avoid potential problems with sparse data.

Socio-demographic variables included sex (male/female), highest level of maternal and paternal education, as well as migrant status. Data on patients’ sex were gathered from the electronic health records. Given the well-known socio-economic disparities in mortality rates [39], also identified among Swedish adolescents [30], we included maternal (including adoptive mothers) as well as paternal (including adoptive fathers) education as indicators of socio-economic status. This was gathered from Statistic Sweden’s LISA database and concerned the highest level of education completed as of 2011. Maternal/paternal education was categorized into low (less than two years of secondary education), intermediate (three years of secondary education), and high (tertiary education). Data on migrant background were gathered from Statistic Sweden’s multigeneration database and distinguished between those born abroad, those born in Sweden with both parents being born abroad, those born in Sweden with one parent born in Sweden and one abroad, and those born in Sweden with both parents being born in Sweden. We included this variable as prior research shows differential patterns of mortality according to migrant background, where for example second-generation immigrants appear to have an excess mortality risk compared to people born in Sweden by two Swedish born parents [40]. Finally, patient age (in full years) at first contact with MU, as well as calendar year at first contact with MU, were used as control variables.

### Statistical analyses

Percentages and mean values were used in the descriptive part of the study. Survival analysis was used to assess the association between the predictors and all-cause mortality. The follow-up period for those who died was defined as the time between the date at first contact with MU and the death date, and for those who were alive during the entire study period (i.e., right-censored), the follow-up was defined as the time between date at first contact with MU and the end of the study period (31 Dec 2021). This approach of defining the start of the follow-up from first day of treatment has been used in other studies as well [7].

We first calculated crude mortality rates (CMRs) overall and for each predictor. Similar to other research (see e.g. [18]), , these are expressed as the number of deaths per 1000 person years. We then estimated Kaplan-Meier failure functions, overall and by predictors, and tested differences according to predictor levels by a log rank test. For number of outpatient visits, we present quartiles in this part of the analyses. The number of outpatient visits were 2 at the first quartile (25th percentile), 4 at the second (50 percentile), and 13 at the third quartile (75th percentile). Finally, we estimated hazard rates (HRs) using Cox regression. We began with unadjusted models for each predictor, followed by a model adjusting for all predictors simultaneously, and ended with a fully adjusted model that also controlled for patients’ age at first contact, and year at first contact with MU. We also ran corresponding Cox regressions including an interaction term between convictions and number of outpatient visits and whether the patient had received inpatient treatment, respectively. The proportional hazards assumption was tested for each predictor in both the unadjusted and adjusted models using Schoenfeld residuals. None of these tests were statistically significant, so there was no evidence that the proportional hazards assumption was violated.

Statistical analyses were run in Stata, vers 18.5.

## Results

### Descriptive statistics

Descriptive statistics are presented in Table 1. Males comprised around 60% of the sample, and the majority were born in Sweden to Swedish born parents (61.44%). Mothers to patients overall had higher education compared to fathers, with close to 50% of the fathers having less than two years of secondary education. A quarter of the sampled individuals had received inpatient treatment, and over 40% had ever been convicted of a criminal offense.

During the study period, 229 individuals died, corresponding to 0.97% of the patients in the cohort. Compared to patients who remained alive throughout the study window, there was a higher proportion of males among deceased patients (75.98% vs. 60.26%). Deceased patients had more outpatient visits (17.33 vs. 11.89) and a larger share had received inpatient treatment (35.81% vs. 24.90%). Deceased patients were also more likely to have ever been convicted of a crime (73.80% vs. 43.57%). No significant differences were observed between deceased and alive patients regarding migrant background, but mothers and fathers of deceased patients had significantly lower education compared to mothers and fathers of alive patients.

Age of death among deceased patients raged from 14 to 32, with an average of 21.64 years (median 21 years, not shown in Table 1). The ultimate cause of death was drug-related for 91 patients (39.74% of all deaths), with drug-related accidents being the most common. One death was alcohol-related. Ultimate causes not related to drug or alcohol use were also relatively common, particularly suicides, accounting for about a quarter of all deaths, but there was also a relatively large number of homicides.

**Table 1:** Descriptive statistics

	All (*n* = 23709)		Alive (*n* = 23480)		Deceased (*n* = 229)		*p*
	*n*	M (sd)/%	*n*	M (sd)/%	*n*	M (sd)/%	
Sex							
Female	9385	35.58	9330	39.74	55	24.02	
Male	14,324	60.42	14,150	60.26	174	75.98	< 0.001
Migrant background							
Born abroad	1461	6.16	1443	6.15	18	7.86	
Born in Sweden, both parents born abroad	3431	14.47	3398	14.47	33	14.41	
Born in Sweden, one parent born abroad	4251	17.93	4209	17.93	42	18.34	
Born in Sweden, both parents born in Sweden	14,566	61.44	14,430	61.46	136	59.39	0.738
Maternal education							
< 2 Yrs upper secondary school	9037	38.12	8931	38.04	106	46.29	
3 Yrs secondary	4399	18.55	4365	18.59	34	14.85	
Tertiary	10,273	43.33	10,184	43.37	89	38.86	0.033
Paternal education							
<2 Yrs upper secondary school	11,214	47.30	11,081	47.19	133	58.08	
3 Yrs secondary	3828	16.15	3793	16,15	35	15.28	
Tertiary	8667	36.56	8606	36.65	61	26.64	0.002
No of outpatient visits	23,709	11.94 (20.95)	23,480	11.89 (20.89)	229	17.33 (25.88)	< 0.001
Inpatient	5928	25.00	5846	24.90	82	35.81	< 0.001
Ever convicted	10,400	43.87	10,231	43.57	169	73.80	< 0.001
Age at death (range 14 to 32)					229	21.64 (3.19)	
Ultimate cause of death							
Drug-related, accident					62	27.07	
Drug-related, suicide					14	6.11	
Drug-related, undetermined intent					15	6.55	
Alcohol-related, accident					1	0.44	
Suicide					57	24.89	
Homicide					40	17.47	
Other cause					40	17.47	

### Survival analysis

Table 2 presents CMRs overall and by predictor variables. The overall CMR was 1.68 deaths per 1000 person years (95% CI = 1.47–1.91), with some important differences between subgroups. In particular, males, patients who had received most outpatient treatment (quartile 4), those who had received inpatient treatment and convicted patients all had excess CMRs. Patients born abroad also had a higher CMR than those with other migrant backgrounds, and those with parents with low level of education had a higher CMR than those whose parents had higher education.

**Table 2:** Crude mortality rates per 1,000 person years, overall and by predictors

	Mortality rates per 1000 person years			
	Deaths (*n*)	Person-years (in units of 1000)	Rate	95%CI
Sex				
Female	55	53.29	1.03	0.79,1.34
Male	174	83.32	2.09	1.80,2.42
Country of birth				
Born abroad	18	8.71	2.07	1.30,3.28
Born in Sweden, both parents born abroad	33	19.10	1.73	1.23,2.43
Born in Sweden, one parent born abroad	42	24.23	1.73	1.28,2.35
Born in Sweden, both parents born in Sweden	136	84.56	1.61	1.36,1.90
Maternal education				
< 2 Yrs upper secondary school	106	56.42	1.88	1.55,2.27
3 Yrs secondary	34	23.85	1.43	1.02,1.99
Tertiary^a^	89	56.33	1.58	1.28,1.95
Paternal education				
<2 Yrs upper secondary school	133	68.71	1.94	1.63,2.29
3 Yrs secondary	35	20.59	1.70	1.22,2.37
Tertiary^a^	61	47.30	1.29	1.00,1.66
Number of outpatient visits (quartiles)				
Qrt1	74	53.38	1.39	1.10,1.74
Qrt2	20	15.02	1.33	0.86,2.06
Qrt3	48	33.00	1.45	1.10,1.93
Qrt4	87	35.21	2.47	2.00,3.05
Inpatient treatment				
No	147	100.32	1.47	1.25,1.72
Yes	82	36.29	2.26	1.82,2.81
Convicted				
No	60	68.90	0.87	0.68,1.12
Yes	169	67.69	2.50	2.15,2.90
Overall	229	136.60	1.68	1.47,1.91

The total time-at-risk was 136,602 years, with an average of 5.76 contributed years (median = 5.79). Kaplan-Meier failure functions, overall and by predictors, are presented in Supplementary Figs. 1a to 1h (95% confidence intervals are omitted for clarity). There were significant differences in the failure curves according to sex, paternal education, number of outpatient visits, inpatient treatment and convictions, but not according to patients’ and parents’ country of birth and maternal education.

Table 3 presents unadjusted and adjusted Cox regression models. Males had significantly higher hazard rates (HRs) than females across unadjusted and adjusted models, ranging from 100% higher in the unadjusted model to about 70% higher in the fully adjusted model. No significant differences according to migrant background or according to maternal education were found in any model. In contrast to what was the case for maternal education, patients with fathers with tertiary education had a significantly lower HR (hazard ratio) compared to those whose fathers had less than two years of secondary education, and the association was similar across models (HRs: 0.67–0.71). The HRs rose by number of outpatient treatments, but this was not significant in any of the adjusted models. Those who had ever received inpatient treatment had between 52 and 89% higher HR compared to those who had received outpatient treatment only, with the associations being stronger in the adjusted models. Patients who had ever been convicted had substantially elevated HRs compared to non-convicted patients, ranging from 183% higher in the unadjusted model to 147% and 164% higher in the adjusted and in the fully adjusted model, respectively.

The lower panel in Table 3 also presents interactions between convictions and number of outpatient visits and having received inpatient treatment. The interaction terms for convictions with number of outpatient visits and inpatient treatment ever were significant but had opposite signs: convicted patients with a higher number of outpatient visits had a smaller HR compared to convicted patients with smaller number of outpatient visits, and convicted patients who had also received inpatient treatment had higher HR compared with convicted patients who had not received inpatient treatment. In the fully adjusted model, the HR for patients who had ever been convicted decreased by a factor of 0.98 for each extra outpatient visit. Also, in the fully adjusted model, convicted patients who had also received inpatient treatment had more than twice the mortality risk (2.50*2.14 = 5.35) compared to convicted patients who had not received inpatient treatment (2.50).

**Table 3:** Hazard ratios and 95% confidence intervals from Cox regression

	Unadjusted		Adjusted		Fully adjusted	
	HR	95%CI	HR	95%CI	HR	95%CI
Sex						
Female	1		1		1	
Male	2.02***	1.50, 2.74	1.71***	1.25,2.35	1.69***	1.23,2.32
Country of birth						
Born abroad	1		1		1	
Born in Sweden, both parents born abroad	0.84	0.48,1.50	0.80	0.45,1.42	0.80	0.45,1.42
Born in Sweden, one parent born abroad	0.84	0.49,1.46	0.86	0.49,1.50	0.88	0.51,1.54
Born in Sweden, both parents born in Sweden	0.78	0.48,1.28	0.85	0.52,1.40	0.84	0.51,1.38
Maternal education						
< 2 Yrs upper secondary school	1		1		1	
3 Yrs secondary	0.77	0.52,1.13	0.84	0.57,1.24	0.86	0.59,1.28
Tertiary^a^	0.85	0.64,1.13	1.10	0.81,1.50	1.10	0.81,1.50
Paternal education						
<2 Yrs upper secondary school	1		1		1	
3 Yrs secondary	0.89	0.61,1.29	0.97	0.67,1.41	0.97	0.66,1.41
Tertiary	0.67**	0.50,0.91	0.71*	0.51,1.0	0.71*	0.51,0.99
No of outpatient visits	1.01**	1.00-1.01	1.00	1.00,1.01	1.00	1.00,1.01
Inpatient treatment						
No	1		1		1	
Yes	1.52**	1.16,2.00	1.76***	1.33,2.32	1.89***	1.42,2.52
Convicted						
No	1		1		1	
Yes	2.83***	2.11,3.80	2.47***	1.80,3.39	2.64***	1.92,3.63
Interactions^a^						
No of outpatient visits	1.01***	1.01,1.02	1.01***	1.01,1.02	1.01***	1.01,1.02
Convicted	3.43***	2.48,4.76	2.48***	1.67,3.66	2.50***	1.69,3.72
No of outpatient visits X Convicted	0.99***	0.98,0.99	0.98***	0.97,0.99	0.98***	0.98,0.99
Inpatient treatment	1.08	0.63,1.86	1.09	0.63,1.89	1.13	0.64,1.97
Convicted	2.37***	1.65,3.40	-	-	-	
Inpatient treatment X Convicted	1.80	0.96,3.37	2.07*	1.10,3.92	2.14*	1.13,4.05
Number of observations	23,709		23,709		23,709	

Fully adjusted model controls for patient age at first contact with MU and year at first contact with MU. HR= hazard ratio; 95%CI = 95% confidence interval; * p ≤ 0.05, **p ≤ 0.01, *** p ≤ 0.001

^a^Adjusted and unadjusted interaction models are controlled for the other variables in the upper panel. Convictions are included twice in the unadjusted models because of two separate models with interactions

## Discussion

This study aimed to examine mortality among patients who had been enrolled at Sweden’s largest regional provider of specialized substance use treatment for adolescents and young adults aged 13 to 25 years, focusing on the period 2011 to 2021. During this time window, 229 individuals died, corresponding to 0.97% of all patients. This figure differs markedly from mortality rates in Sweden for individuals in similar age brackets of the general population, although direct comparisons are difficult to make because of different ages and years at first contact with MU across the sample (e.g., older patients who entered treatment in the beginning of the period could be in their thirties at the end of the period). Between 2011 and 2021, the morality rate per 1,000 individuals in the general Swedish population was between 0.20 and 0.28 for individuals aged 15–19 years, 0.38–0.48 for 20–24-year-olds, 0.45–0.55 for 25-29-year-olds, 0.49–0.62 for 30-34-year-olds, and 0.56–0.67 for those aged 35–39 years [41]. The mortality rate in our study (corresponding to 9.7 per 1000 individuals) is thus between approximately 14 to 49 times than the highest (0.67) and lowest (0.20) of these population benchmarks. These findings are consistent with prior research showing profoundly higher mortality rates in substance use treatment samples compared to the general population [7–11, 13, 14], particularly among younger individuals [10], and illustrate that people in treatment is a highly disadvantaged group overall with a substantial unmet need for healthcare and social interventions.

We found that the most common cause of death was drug-related accidents (27.07%), but suicides not related to drugs (24.89%) were almost as common, and a relatively large share died from homicides (17.47%). Deaths where drugs and alcohol were the ultimate causes accounted for about 40% of the deaths. This figure is similar to what was found in an Australian study by Bista et al. [18], although they addressed youth in residential treatment. However, the crude mortality rate (CMR) per 1,000 person years was lower in our study compared to theirs, and we also observed larger differences in CMRs between males and females compared to what they did. The differences in the overall CMRs are probably related to the fact that the vast majority of the patients in our study did not receive inpatient treatment, and as such were not as heavily burdened. Here it should be mentioned that all adolescents in Sweden who are caught using illegal drugs – including cannabis - are referred to treatment more or less regardless of how much they use [42]. Thus, our sample probably also include relatively well-off cannabis testers [43], which may account for some of the differences in mortality rates between Sweden and countries with different cannabis policies. Supporting these considerations, the CMR for those who received inpatient treatment in our sample was higher compared to those who had received outpatient treatment only. However, higher CMRs than those observed here have been found in outpatient treatment samples in the US (see [15]), suggesting important national differences in CMRs also among outpatients across countries. Again, this may be due to variations in the compositions of the treatment populations in different settings.

We observed some clear patterns in the associations between the predictors included and mortality. Being male, having received inpatient treatment and having been convicted for a criminal offense were all associated with a significantly higher HR for all-cause mortality. The results pertaining to sex are congruent with some studies (e.g [15]), but not with others, where the differences in mortality have either been relatively small [18], or not statistically significant [24]. The reasons behind these conflicting findings are not easily pinpointed, but may be related to differences in year cohorts, follow-up times, national contexts, and variables adjusted for.

There was an excess HR among patients who were convicted during the period, with a fully adjusted HR that was over 160% higher compared to non-convicted patients. This finding is consistent with prior studies [10, 35, 44], and suggests that criminality is a strong correlate of mortality among adolescents in substance use treatment. Deaths due to external causes such as intoxication and violence are more common in younger as opposed to older age groups in treatment, where somatic issues are more pertinent, and mortality risks are especially elevated among young, convicted patients in general and patients convicted for violent offenses in particular [10]. The high prevalence of convictions in the sample (> 40%), coupled with the risks that are related to a criminal lifestyle, may thus be an important driver behind the high mortality rate observed. Prior research points to poorer health as a factor behind excess mortality among offenders [35], and the relatively high prevalence of suicides and homicides among deceased patients highlights the importance of considering a range of factors to make sense of the link between offending and mortality. While suicides are likely to be related to psychiatric problems, or despair more generally (e.g [30]), homicides can at least for males be assumed to be more strongly related to conflicts between rival gangs on the drug market. The rising levels of firearm shootings observed in Sweden in recent years, a trend that departs from what is the case in other European countries, have largely affected young men affiliated with criminal gangs [45]. As it seems, this is also reflected among a non-trivial share of deceased patients. Combined, the various causes of deaths point to the need for a comprehensive approach for preventing premature deaths among patients in substance use treatment, also including actors such as child and adolescent psychiatry and prison and probation services. Aftercare is also likely to be a crucial component for some patient groups [17]. For example, research on people admitted to inpatient psychiatric care shows that the risk of suicide is particularly pronounced the first months after discharge [46], indicating that immediate time after treatment is a critical factor to consider.

Whether the observed association between convictions and mortality is causal cannot be determined using the research design employed in the current study. It is plausible that convictions do have a direct or indirect effect on mortality, but the association may also be spurious [47]. However, the HRs for convictions were relatively similar in the unadjusted models and the models adjusting for potential confounders (e.g., parental socio-economic status [SES], migrant background), meaning that convictions are unlikely to only be markers of socioeconomic disparities in mortality. We find it likely, though, that at least some offenses may proxy for other risk behaviors [10], and convictions related to drug offenses may serve as proxy measures for severity of substance use problems, and/or concurrent social problems.

We also explored how treatment experience was related to mortality. Number of outpatient visits were positively associated with mortality in the unadjusted but not in the adjusted models. This is in contrast to what was the case for inpatient treatment, where patients who had received inpatient treatment at least once had an excess HR of 89% in the fully adjusted model. Fleury et al. [32], using a sample of substance use treatment patients in Canada, observed lower risk of physical as well as accidental or intentional deaths in patients who had received more consultations with psychiatrists and who had received more interventions within substance use treatment. However, they identified a reverse pattern for number of visits to emergency departments, and those who had been hospitalized (for any reason) also had an excess mortality risk. A similar logic is likely to hold true for inpatients in this study: at MU Stockholm, inpatient treatment can be both acute and planned, and may as such be provided both to those who are acutely intoxicated or otherwise have more extensive problems. As those who receive more outpatient treatment are also more likely to receive inpatient treatment, this may account for the non-significant association between number of outpatient visits and mortality in the adjusted models.

We also tested for statistical interactions between convictions and treatment received. While the main effects analyses showed excess mortality risks for those who had received inpatient treatment, these analyses identified opposite associations for outpatient treatment versus inpatient treatment and convictions. A higher number of outpatient treatment episodes among convicted patients decreased the mortality risk, but the opposite was found for inpatient treatment. The mortality risk for convicted patients who had also received inpatient treatment was over two times higher compared to convicted patients who had not received inpatient treatment. Although these results are to be considered exploratory, they do suggest that criminality and treatment experiences may interact in complex ways. Convicted patients subjected to inpatient treatment may differ in criminal profile as well as in substance use and psychiatric problems compared to convicted patients who only receive outpatient care (cf [18]), and such potential differences may drive the higher mortality risk among the former. Future research should examine if these patterns can be observed in other samples of adolescents and young adults in substance use treatment, and if they remain when adjusting for additional substance use, psychiatric and criminality-related factors.

Of note, we found no clear evidence for a graded mortality risk according to migrant background. These findings disagree with general population, register based studies on mortality in Sweden [40]. In part, this may be due to the fact that we used quite crude variables and did not consider factors such as age of arrival in Sweden for foreign-born patients, or parents’ country of birth for patients whose parents were born abroad. However, given the sample, some factors that may fuel different mortality rates according to migrant status in normal population studies (e.g., psychiatric problems, substance use) are probably to some extent controlled for by design in this study, which in turn could attenuate the association between migrant background and mortality. For example, it is widely known that psychiatric problems are common among people in substance use treatment [23]. There was a general lack of significant differences regarding socio-economic status (SES). These findings were unexpected given well-established socio-economic differences in mortality rates [39], increasing disparities in convictions between different socio-economic groups [48], and in deadly violence among young people affiliated with criminal gangs in Sweden [27]. We believe that similar considerations as for migrant status apply for SES: many factors that may account for SES differences in general population samples are controlled for by design in treatment samples where there is a higher concentration of psychiatric and other problems. It should also be noted that not only do our findings depart from the frequently observed excess mortality risk among groups with lower SES – a recent study on adult substance users in contact with the social services in Sweden found that individuals with higher educational attainment were more likely to die from alcohol and drug related causes [21]. This suggests that people with higher SES in treatment is a select group not representative of its counterpart in the general population [21]. Thus, we emphasize that these findings do not challenge the well-known link between SES and mortality in general [39]; moreover, the relatively short follow-up window should also be mentioned here, as potential disparities in mortality between different SES groups likely aggregate as individuals age and all-cause mortality increases.

### Strengths and limitations

Some important limitations should be mentioned. First, by focusing on all-cause mortality, our regression models have not addressed the potential of differential associations between the predictors and cause-specific mortality [36]. Second, our measure of convictions was quite crude, and did not distinguish between different crimes involved in the convictions. As only a share of offenses actually committed are included in the registers, the prevalence of criminality in the sample is underestimated. Third, we lacked data on the extent of substance use problems, which is likely an important predictor of mortality. Nonetheless, the sample comprises only patients who were in treatment because of their substance use, which would not be the case in studies on other clinical or general population samples. In fact, although the mortality risk differs across substances used in treatment samples, excess SMRs have been observed among many different illegal substances used in these samples (e.g., cannabis, amphetamine, heroin [7]), .

Key strengths of the study include the large sample size, and the high coverage of patients admitted to Sweden’s largest regional provider of specialized substance use treatment to adolescents for over a decade. Our use of data from patient journals and Swedish registers is also a strength, as the registers have a high coverage and avoid problems with self-reported data. In contrast to prior Swedish mortality studies on adolescents in treatment [24], our data also cover a relatively recently treated patient population. This means that the findings are highly relevant for the provision of substance use treatment for contemporary adolescents and young adults.

## Conclusion

Close to 1% of all patients who were in contact with MU Stockholm between 2011 and 2021 died during this period. Being male, having received inpatient treatment, and having been convicted of a criminal offense were associated with an excess mortality rate. No clear associations with mortality were found for migrant background, parental SES, or amount of outpatient treatment. Our study suggests that there may be an important interaction between criminality and substance use treatment that should be explored further in future studies on mortality rates in clinical populations.

## Supplementary Information

Below is the link to the electronic supplementary material.

Supplementary Material 1.

Supplementary Material 2.

## Data Availability

The data sets generated and analyzed in this study are not publicly available due to patient confidentiality and cannot be shared without permission from the healthcare provider; contact the corresponding author for more information on how to obtain the data.

## References

[1] Erskine HE, Moffitt TE, Copeland WE, Costello EJ, Ferrari AJ, Patton G, Degenhardt L, Vos T, Whiteford HA, Scott JG. A heavy burden on young minds: the global burden of mental and substance use disorders in children and youth. Psychol Med. 2015;45:1551–63. 25534496 10.1017/S0033291714002888 PMC5922255

[2] Hermans SP, Samiec J, Golec A, Trimble C, Teater J, Hall OT. Years of Life Lost to Unintentional Drug Overdose Rapidly Rising in the Adolescent Population, 2016–2020. J Adolesc Health. 2023;72:397–403. 36096899 10.1016/j.jadohealth.2022.07.004

[3] Friedman J, Hadland SE. The Overdose Crisis among U.S. Adolescents. N Engl J Med. 2024;390:97–100. 38198189 10.1056/NEJMp2312084 PMC10979414

[4] Ågren G, Bremberg S. Mortality trends for young adults in Sweden in the years 2000–2017. Scand J Public Health. 2022;50:448–53. 33764225 10.1177/14034948211000836 PMC9152592

[5] Socialstyrelsen. Statistik om dödsorsaker år 2024. Stockholm: Socialstyrelsen; 2025.

[6] EUDA. European Drug Report. 2025. Trends and developments. European Union Drugs Agency; 2025.

[7] Arendt M, Munk-Jørgensen P, Sher L, Jensen SO. Mortality among individuals with cannabis, cocaine, amphetamine, MDMA, and opioid use disorders: a nationwide follow-up study of Danish substance users in treatment. Drug Alcohol Depend. 2011;114:134–9. 20971585 10.1016/j.drugalcdep.2010.09.013

[8] Engqvist U, Rydelius PA. Death and suicide among former child and adolescent psychiatric patients. BMC Psychiatry. 2006;6:51. 17081290 10.1186/1471-244X-6-51 PMC1635416

[9] Huỳnh C, Kisely S, Rochette L, Pelletier É, Morrison KB, Li S, Hopkin G, Smith M, Burchill C, Lin E, Asbridge M, Jutras-Aswad D, Lesage A. Measuring Substance-Related Disorders Using Canadian Administrative Health Databanks: Interprovincial Comparisons of Recorded Diagnostic Rates, Incidence Proportions and Mortality Rate Ratios. Can J Psychiatry. 2022;67:117–29. 34569874 10.1177/07067437211043446 PMC8978214

[10] Jakobsson J, Karlsson A, Håkansson A, Hofvander B. Mortality among individuals with substance use disorder-does violent criminal behavior have an impact? Front Psychiatry. 2024;15:1455343. 39713773 10.3389/fpsyt.2024.1455343 PMC11659145

[11] James A, Clacey J, Seagroatt V, Goldacre M. Adolescent inpatient psychiatric admission rates and subsequent one-year mortality in England: 1998–2004. J Child Psychol Psychiatry. 2010;51:1395–404. 20738446 10.1111/j.1469-7610.2010.02293.x

[12] Lindblad R, Hu L, Oden N, Wakim P, Rosa C, VanVeldhuisen P. Mortality Rates Among Substance Use Disorder Participants in Clinical Trials: Pooled Analysis of Twenty-Two Clinical Trials Within the National Drug Abuse Treatment Clinical Trials Network. J Subst Abuse Treat. 2016;70:73–80. 27692192 10.1016/j.jsat.2016.08.010 PMC5117359

[13] Prestmo A, Høyen K, Vaaler AE, Torgersen T, Drange OK. Mortality Among Patients Discharged From an Acute Psychiatric Department: A 5-Year Prospective Study. Front Psychiatry. 2020;11:816. 33013492 10.3389/fpsyt.2020.00816 PMC7461827

[14] Zábranský T, Csémy L, Grohmannová K, Janíková B, Brenza J. Mortality of cohort of very young injecting drug users in Prague, 1996–2010. Cent Eur J Public Health. 2011;19(3):152–7. 22026292 10.21101/cejph.a3681

[15] Thurstone C, Etzig C, Chen E, Seely HD, Loh R. Mortality following adolescent substance treatment: 21-year follow-up from a single clinical site. Front Child Adolesc Psychiatry. 2025;4:1600101. 40620525 10.3389/frcha.2025.1600101 PMC12226572

[16] Chesney E, Goodwin GM, Fazel S. Risks of all-cause and suicide mortality in mental disorders: a meta-review. World Psychiatry. 2014;13(2):153–60. 24890068 10.1002/wps.20128 PMC4102288

[17] Pitkänen T, Laine R, Kaskela T, Uusimäki V, Levola JA, Register-Based. Follow-Up Study of Age-Specific Mortality and Causes of Death of Men, Women and Accompanying Children After Substance Use Treatment. Nordisk Alkohol Nark. 2025;42:514–31. 41180473 10.1177/14550725251388250 PMC12578615

[18] Bista S, Nathan S, Rawstorne P, Palmer K, Ferry M, Williams M, Hayen A. Mortality among young people seeking residential treatment for problematic drug and alcohol use: A data linkage study. Drug Alcohol Depend. 2021;228:109030. 34592701 10.1016/j.drugalcdep.2021.109030

[19] Fugelstad A, Annell A, Ågren G. Long-term mortality and causes of death among hospitalized Swedish drug users. Scand J Public Health. 2014;42:364–9. 24608092 10.1177/1403494814525006

[20] von Greiff N, Skogens L, Berlin M, Bergmark A. Mortality and Cause of Death-A 30-Year Follow-Up of Substance Misusers in Sweden. Subst Use Misuse. 2018;53:2043–51. 29578830 10.1080/10826084.2018.1452261

[21] Scarpa S, Jemberie WB, Högberg B, Lundgren L. Educational attainment and deaths of despair among individuals assessed for substance use severity: Findings from Swedish Addiction Severity Index (ASI) data. Nordisk Alkohol Nark. 2025;42:479–98. 40094100 10.1177/14550725251326757 PMC11907572

[22] Christie GI, Bavin LM, Wills S. Can We Predict Which Adolescents Will Engage in Outpatient Substance Abuse Treatment? Subst Abuse. 2018;12:1178221818762802. 29568221 10.1177/1178221818762802 PMC5858732

[23] Levola J, Laine R, Pitkänen T. In-patient psychiatric care and non-substance-related psychiatric diagnoses among individuals seeking treatment for alcohol and substance use disorders: associations with all-cause mortality and suicide. Br J Psychiatry. 2022;221:386–93. 35164892 10.1192/bjp.2022.20

[24] Larm P, Hodgins S, Larsson A, Samuelson YM, Tengström A. Long-term outcomes of adolescents treated for substance misuse. Drug Alcohol Depend. 2008;96:79–89. 18375076 10.1016/j.drugalcdep.2008.01.026

[25] Molero Samuelson Y, Hodgins S, Larsson A, Larm P, Tengström A. Adolescent antisocial behavior as predictor of adverse outcomes to age 50: A follow-up study of 1,947 individuals. Crim Justice Behav. 2010;37:158–74.

[26] CAN. CAN:s nationella skolundersökning 2025. Ungas erfarenheter av alkohol, narkotika, dopning, tobak och spel om pengar. Stockholm: Centralförbundet för alkohol- och narkotikaupplysning; 2025.

[27] Khoshnood A. Firearm-related violence in Sweden–A systematic review. Aggress Violent Behav. 2018;42:43–51.

[28] Ekendahl M, Månsson J, Karlsson P. Media constructions of an illegal drug: The link between cannabis and organized crime in Swedish newspapers. Drugs Educ Prev Policy. 2024;31:300–9.

[29] Case A, Deaton A. Rising morbidity and mortality in midlife among white non-Hispanic Americans in the 21st century. Proc Natl Acad Sci U S A. 2015;112:15078–83. 26575631 10.1073/pnas.1518393112 PMC4679063

[30] Högberg B, Scarpa S, Petersen S. Trends in educational inequalities in all-course mortality and deaths of despair in Swedish youths 1990–2018. SSM Popul Health. 2025;29:101748. 39886260 10.1016/j.ssmph.2025.101748 PMC11780141

[31] King L, Scheiring G, Nosrati E. Deaths of despair in comparative perspective. Annu Rev Sociol. 2022;48:299–317.

[32] Fleury MJ, Cao Z, Grenier G, Huỳnh C. Predictors of Death From Physical Illness or Accidental/Intentional Causes Among Patients With Substance-Related Disorders. Can J Psychiatry. 2023;68:163–77. 36317322 10.1177/07067437221136461 PMC9974654

[33] Elonheimo H, Sillanmäki L, Sourander A. Crime and mortality in a population-based nationwide 1981 birth cohort: Results from the FinnCrime study. Crim Behav Ment Health. 2017;27:15–26. 26307464 10.1002/cbm.1973

[34] Piquero AR, Farrington DP, Shepherd JP, Auty K. Offending and early death in the Cambridge Study in Delinquent Development. Justice Q. 2014;31:445–72.

[35] Skinner GC, Farrington DP. A systematic review and meta-analysis of premature mortality in offenders. Aggress Violent Behav. 2020;53:101431.

[36] Olsson MO, Bradvik L, Öjehagen A, Hakansson A. Risk factors for unnatural death: Fatal accidental intoxication, undetermined intent and suicide: Register follow-up in a criminal justice population with substance use problems. Drug Alcohol Depend. 2016;1:162:176–81. 10.1016/j.drugalcdep.2016.03.009 27020324

[37] Romero D, Kåberg M, Berman AH, Carlbring P, Franck J, Lindner P. Predictors of outpatient treatment engagement following a visit to a specialized emergency department for substance use: A cohort study using high-resolution electronic health records. Addiction. 2026;121:1237–48. 41582065 10.1111/add.70335 PMC13088932

[38] Karlsson P, Ekendahl M, Lindner P. How Much Treatment are Adolescents Receiving in Specialised Substance Use Healthcare in Sweden? Age and Cohort Trends. Subst Use Res Treat. 2025;19:29768357251351758. 10.1177/29768357251351758 PMC12227927 40620485

[39] Bosworth B. Increasing Disparities in Mortality by Socioeconomic Status. Annu Rev Public Health. 2018;39:237–51. 29608870 10.1146/annurev-publhealth-040617-014615

[40] Wallace M. Mortality Advantage Reversed: The Causes of Death Driving All-Cause Mortality Differentials Between Immigrants, the Descendants of Immigrants and Ancestral Natives in Sweden, 1997–2016. Eur J Popul. 2022;38(5):1213–41. 36507238 10.1007/s10680-022-09637-0 PMC9727037

[41] SCB. Deaths by region, age (during the year) and sex. Year 1968–2024. https://www.statistikdatabasen.scb.se/pxweb/sv/ssd/START__BE__BE0101__BE0101I/DodaHandelseK/ (accessed 2026-01-20). Statistiska Centralbyrån: Statistical Database;2026.

[42] Ekendahl M, Kvarmans P, Karlsson P. Urine samples and drug-body-treatment assemblages: Youth enactments of drug testing in Sweden. Contemp Drug Probl. 2024;51:163–77.

[43] Karlsson P, Ekendahl M, Månsson J, Raninen J. Has illicit drug use become normalised in groups of Swedish youth? A latent class analysis of school survey data from 2012 to 2015. Nordisk Alkohol Nark. 2019;36:21–35. 32934547 10.1177/1455072518814306 PMC7434166

[44] Stenbacka M, Moberg T, Jokinen J. Adolescent criminality: multiple adverse health outcomes and mortality pattern in Swedish men. BMC Public Health. 2019;19:400. 30975117 10.1186/s12889-019-6662-z PMC6460509

[45] Hradilova Selin K, Krüsselmann K, Suonpää K, Shannon D. (2024). Trends in firearm homicide in 23 European countries–is Sweden an outlier? Nord J Criminol. 2024; 25: 1–24.

[46] Chung DT, Ryan CJ, Hadzi-Pavlovic D, Singh SP, Stanton C, Large MM. Suicide Rates After Discharge From Psychiatric Facilities: A Systematic Review and Meta-analysis. JAMA Psychiatry. 2017;74:694–702. 28564699 10.1001/jamapsychiatry.2017.1044 PMC5710249

[47] van de Weijer S, Bijleveld C, Huschek D. Offending and mortality. Adv Life Course Res. 2016;28:91–9.

[48] Nilsson A, Estrada F, Bäckman O. The unequal crime drop: Changes over time in the distribution of crime among individuals from different socioeconomic backgrounds. Eur J Criminol. 2017;14:586–605.

