# Supplementary material for: Mortality among adolescent and young adults in specialized substance use treatment: a Swedish register study
Source: Child Adolesc Psychiatry Ment Health. 2026 Jun 24;20:92. doi: 10.1186/s13034-026-01125-1 (PMC13317170; doi:10.1186/s13034-026-01125-1)
Supplement: Supplementary file 1 — Supplementary Material 1. [file 13034_2026_1125_MOESM1_ESM.docx]

**Supplementary file 1**

All causes of death during 2011 to 2021 were coded according to the Swedish version of ICD-10 (ICD-10-SE). Our data differ in detail across causes of death, with specific codes being available for deaths due to conditions included in chapter 19 in ICD-10 (“Injury, Poisoning, and Certain Other Consequences of External Causes”). Codes for specific causes of these deaths were obtained from chapter 20 in ICD-10 (“External causes of morbidity and mortality”). Data on other causes only consist of the range of codes in a specific chapter in ICD-10 in which the death code is included. In the results, these are referred to as “other causes”.

Following the National Board of Health and Welfare[1], we defined deaths as *directly* caused by drugs (including medicines) if the ultimate cause included the codes X40-X44 (accidents), X60-X64 (suicides) and Y10-Y14 (undetermined intent). Note that this definition of drug-related deaths is more inclusive than the definition used by the European Union Drugs Agency (EUDA) as it also includes deaths related to medicines, which is also the case in the US [1]. We defined deaths as directly related to alcohol if the ultimate cause included the code X45 (suicide), X65 (accidents), and Y15 (undetermined intent). Deaths were defined as suicides (not drug or alcohol-related) if the ultimate cause included the codes X66 to X84, as homicides if the ultimate cause included the codes X85-Y09, and as accidents (not drug or alcohol-related) if the ultimate cause included the codes Y16-Y34.

**References**

1. Socialstyrelsen. Dödsfall till följd av läkemedels- och narkotikaförgiftningar. En statistiksammanställning: Stockholm: Socialstyrelsen;2022
