# Supplementary material for: Mortality among adolescent and young adults in specialized substance use treatment: a Swedish register study
Source: Child Adolesc Psychiatry Ment Health. 2026 Jun 24;20:92. doi: 10.1186/s13034-026-01125-1 (PMC13317170; doi:10.1186/s13034-026-01125-1)
Supplement: Supplementary file 2 — Supplementary Material 2. [file 13034_2026_1125_MOESM2_ESM.docx]

**Supplementary file 2**

*Fig 1a. Kaplan-Meier failure function, overall*

**

*Fig 1b.* *Kaplan-Meier failure function, by sex (p<0.001, logrank test)*

*Fig 1c. Kaplan-Meier failure function, by patient’s and parents’ country of birth (p=0.786, logrank test)*

*Fig 1d. Kaplan-Meier failure function, by maternal education (p=0.319, logrank test)*

*Fig 1e. Kaplan-Meier failure function, by paternal education (p=0.036, logrank test)*

*Fig 1f. Kaplan-Meier failure function, by number of outpatient visits (quartiles)(p<0.001, logrank test)*

**

*Fig 1g. Kaplan-Meier failure function, by inpatient treatment (p=0.002, logrank test)*

*Fig 1h. Kaplan-Meier failure function, by conviction (p<0.001, logrank test)*
